# Supplementary material for: Precise Authenticity of Quinoa, Coix Seed, Wild Rice and Chickpea Components Using Optimized TaqMan Real-Time PCR
Source: Foods. 2023 Feb 16;12(4):852. doi: 10.3390/foods12040852 (PMC9957468; doi:10.3390/foods12040852)
Supplement: Supplementary file 1 [file foods-12-00852-s001.zip › foods-2172926-supplementary.pdf]

Supporting Information

Article

Precise and rapid authenticity of functional food components using optimized TaqMan real-time quantitative PCR

Qiuyue Zheng[1,†], Xinying Yin[1,†], Aifu Yang[2], Ning Yu[3], Ranran Xing[3], Ying Chen[3*], Ruijie Deng[4], Jijuan Cao[1*]

[1] Key Laboratory of Biotechnology and Bioresources Utilization of Ministry of Education, College of Life Science, Dalian Minzu University, Dalian 116600, China

[2] Technology Center of Dalian Customs District, Dalian 116001, China

[3] Chinese Academy of Inspection and Quarantine, Beijing 322001, China

[4] College of Biomass Science and Engineering, Healthy Food Evaluation Research Center, Sichuan University, Chengdu 610065, China

† These authors contributed equally to this work.

* Correspondence: chenyingcaiq@163.com (Y.C.); caojijuan@dlnu.edu.cn (J.C.)

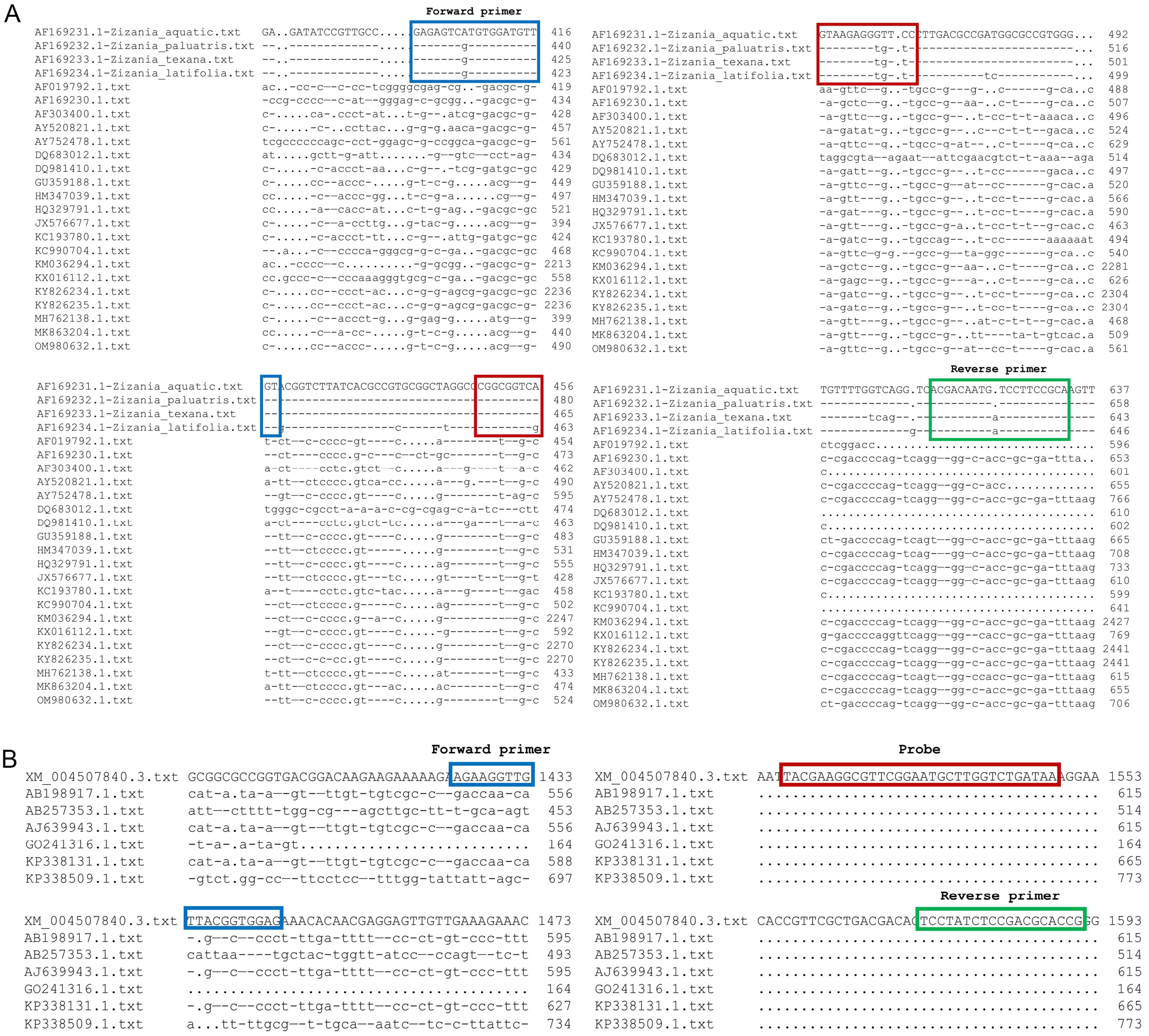

Figure S1. Specific analysis of primer and probe. (A) Specificity comparison of primer and probe sequence for the ITS gene region developed using MEGA 4.0 for the specific qPCR detection of wild rice source component. (B) Specificity comparison of primer and probe sequences for the CIA-2 gene region developed for specific qPCR detection of chickpea source component using MEGA 4.0.

Table S1. Conditions of robustness test as an orthogonal design [a]

Factors	Combination								
	**1**	**2**	**3**	**4**	**5**	**6**	**7**	**8**	**Control**
qPCR instrument	A	A	A	A	B	B	B	B	B
qPCR reagent	X	X	Y	Y	X	X	Y	Y	X
Primer concentration (pmol/μL)	Unchanged	-20%	Unchanged	-20%	Unchanged	-20%	Unchanged	-20%	Unchanged
Probe concentration (pmol/μL)	Unchanged	-20%	-20%	Unchanged	-20%	Unchanged	Unchanged	-20%	Unchanged
Volume of reaction mixture (2×)	13.5 μL	13.5μL	11.5 μL	11.5μL	13.5 μL	13.5μL	11.5 μL	11.5μL	Unchanged
Annealing temperature	+1°C	- 1°C	+1°C	- 1°C	- 1°C	+1°C	- 1°C	+1°C	Unchanged

[a] A : LightCycler® 480 II Real-Time Fluorescence PCR (Roche, Germany). B: QuantStudio6 Flex Real-Time Fluorescence PCR (ABI, USA). X: Premix Ex TaqTM Probe qPCR, RR390 A, TAKARA, China. Y: GoTaq qPCR Master Mix, A6101, Promega, USA.

Table S2. qPCR specific analysis results of quinoa, coix seed, wild rice and chickpea

Samples		Average Ct value				
**Common name**	**Latin name**	**wild rice system**	**quinoa system**	**coix seed system**	**chickpea system**	**18S rRNA**
coix seed	*Coix lacryma-jobi*	negative	negative	22.2 ± 1.02	negative	18.8 ± 0.08
wild rice	*Zizania latifolia*	22.9 ± 0.10	negative	negative	negative	17.4 ± 0.07
wild rice	*Zizania palustris*	23.7 ± 0.69	negative	negative	negative	17.8 ± 0.06
wild rice	*Zizania aquatica*	25.4 ± 0.60	negative	negative	negative	17.3 ± 0.04
wild rice	*Zizania texana*	21.9 ± 0.12	negative	negative	negative	17. 7 ± 0.07
quinoa (white)	*Chenopodium quinoa Willd.*	negative	26.2 ± 1.13	negative	negative	13.6 ± 0.13
quinoa (red)	*Chenopodium quinoa Willd.*	negative	26.1 ± 1.12	negative	negative	15.7 ± 0.07
quinoa (black)	*Chenopodium quinoa Willd.*	negative	26.4 ± 1.14	negative	negative	14.1 ± 0.11
chickpea	*Cicer arietinum L.*	negative	negative	negative	24.3 ± 0.79	16.3 ± 1.98
sorghum rice	*Sorghum*	negative	negative	negative	negative	16.5 ± 0.22
sugarcane	*Saccharum officin*	negative	negative	negative	negative	18.4 ± 0.14
pseudo sorghum rice	*Sorghum halepense (L.) Pers*	negative	negative	negative	negative	18.4 ± 0.15
corn	*Zea mays L.*	negative	negative	negative	negative	15.8 ± 0.11
Chia seed	*Salvia Hispanica L.*	negative	negative	negative	negative	13.9 ± 0.21
red rice	*Lpomoea batatas (L.)*	negative	negative	negative	negative	17.7 ± 0.08
black kerneled rice	*Semen Trigone*	negative	negative	negative	negative	14.8 ± 1.06
rice	*Oryza sativa*	negative	negative	negative	negative	14.5 ± 0.11
coarse rice	*Panicum miliaceum*	negative	negative	negative	negative	14.8 ± 0.07
Rye	*Secale cereale L.*	negative	negative	negative	negative	13.9 ± 2.33
buckwheat	*Fagopyrum esculentum Moench.*	negative	negative	negative	negative	15.0 ± 0.21
tartary buckwheat	*Fagopyrum tataricum (L.) Gaertn.*	negative	negative	negative	negative	16.6 ± 0.14
oats	*Avena sativa L.*	negative	negative	negative	negative	15.1 ± 0.37
barley	*Hordeum vulgare L.*	negative	negative	negative	negative	16.4 ± 0.10
wheat	*Triticum aestivum L.*	negative	negative	negative	negative	15.1 ± 0.05
Semen sesami nigrum	*Sesamum radiatum*	negative	negative	negative	negative	16.6 ± 0.11

black soya bean	*Glycine max (L.) merr.*	negative	negative	negative	negative	14.1	±	0.14
kidney bean	*Phaseolus vulgaris*	negative	negative	negative	negative	14.7	±	0.08
soybean	*Glycine max (Linn.) Merr.*	negative	negative	negative	negative	14.8	±	0.15
Mung bean	*Vigna radiata (Linn.) Wilczek.*	negative	negative	negative	negative	14.4	±	0.07
hyacinth bean	*Lablab purpureus (Linn.) Sweet*	negative	negative	negative	negative	12.8	±	0.02
pea	*Pisum sativum L.*	negative	negative	negative	negative	15.7	±	0.05
red bean	*Vigna angularis*	negative	negative	negative	negative	12.5	±	0.17
agaric	*Auricularia auricula (L.ex Hook.)Unegativeerwood*	negative	negative	negative	negative	14.8	±	0.19
Xianggu mushroom	*Lentinus edodes (Berkeley) Singer*	negative	negative	negative	negative	14.6	±	0.15

Table S3. linear equation of qPCR assay of quinoa, coix seed, wild rice anegative chickpea

Event	Linear equation	R^2	Efficiency (E)%
quinoa source component	y = 27.16 - 3.27 Log10 X	0.9923	102.22%
coix seed source component	y = 26.59 - 3.35 Log10 X	0.9984	98.74%
wild rice source component	y = 23.67 - 3.40 Log10 X	0.9965	96.97%
chickpea source component	y = 26.82 - 3.38 Log10 X	0.9989	97.80%

Table S4. Robustness results of quinoa, coix seed, wild rice and chickpea systems

Methods	Ct value of combination										RSD (%)
	**1**	**2**	**3**	**4**	**5**	**6**	**7**	**8**	**Mean**	**Control**	
Quinoa	35.32 ± 0.11	35.43 ± 0.15	35.45 ± 0.11	35.38 ± 0.15	35.34 ± 0.04	35.36 ± 0.12	35.42 ± 0.15	35.47 ± 0.14	35.40 ± 0.05	35.27 ± 0.05	0.14
Coix seed	35.89 ± 0.14	35.92 ± 0.12	35.99 ± 0.11	35.81 ± 0.16	35.94 ± 0.16	35.92 ± 0.14	36.02 ± 0.14	35.96 ± 0.13	35.93 ± 0.06	35.93 ± 0.08	0.17
Wild rice	32.73 ± 0.14	32.72 ± 0.22	32.66 ± 0.15	32.74 ± 0.20	33.05 ± 0.18	33.01 ± 0.20	32.97 ± 0.11	32.99 ± 0.11	32.86 ± 0.16	32.82 ± 0.07	0.49
Chickpea	35.67 ± 0.10	35.67 ± 0.08	35.80 ± 0.12	35.81 ± 0.11	36.27 ± 0.52	36.00 ± 0.14	36.01 ± 0.19	36.05 ± 0.17	35.91 ± 0.21	35.77 ± 0.05	0.58

Table S5. Detection of commercial samples containing quinoa, coix seed, wild rice and chickpea

Sample No.	Sample name	qPCR analysis results (Ct value)			
		Quinoa	Coix seed	Wild rice	Chickpea
1	Coix seed (100%)	negative	23.2 ± 0.08	negative	negative
2	Red bean coix seed Yam powder	negative	32.3 ± 0.12	negative	negative
3	Coix seed red bean Meal replacement cake	negative	negative	negative	negative
4	Red bean coix seed pellet	negative	negative	negative	negative
5	Red bean coix seed Poria cocos cream	negative	36.2 ± 0.11	negative	negative
6	Red bean coix seed steamed bun	negative	36.0 ± 0.04	negative	negative
7	Red bean coix seed tea	negative	33.4 ± 0.14	negative	negative
8	Red bean coix seed Soft Canegativey	negative	35.8 ± 0.21	negative	negative
9	Coix seed noodle	negative	37.5 ± 0.12	negative	negative
10	Red bean coix seed Poria cocos tea	negative	32.7 ± 0.10	negative	negative
11	Chickpea (100%)	negative	negative	negative	24.6 ± 0.09
12	Chickpea noodle (30%)	negative	negative	negative	27.3 ± 0.30
13	Steamed and ready to eat chickpea chips (100%)	negative	negative	negative	24.2 ± 0.08
14	Refined pure raw chickpea leather (100%)	negative	negative	negative	30.6 ± 0.19
15	Canned Turkish chickpea (100%)	negative	negative	negative	37.8 ± 0.07
16	Canned chickpea (100%)	negative	negative	negative	31.3 ± 0.15
17	Fried chickpea (100%)	negative	negative	negative	36.9 ± 0.21
18	Quinoa Bolivia imports quinoa (100%)	26.0 ± 0.17	negative	negative	negative
19	Qinghai 3 colors quinoa (100%)	26.8 ± 0.22	negative	negative	negative
20	Quinoa soda biscuit (3.2%)	negative	negative	negative	negative
21	Quinoa wheat Germ Mixed Cereal (2%)	36.7 ± 0.57	negative	negative	negative
22	Quinoa powder (100%)	26.2 ± 0.08	negative	negative	negative
23	Canada No.1 Ice lake wild rice (100%)	negative	negative	20.7 ± 0.14	negative
24	Wild rice powder (100%)	negative	negative	22.1 ± 0.04	negative
